# Human Codon Usage: The Genetic Basis of Pathogen Latency

**DOI:** 10.1055/s-0041-1729753

**Published:** 2021-06-14

**Authors:** Darja Kanduc

**Affiliations:** 1 Department of Biosciences, Biotechnologies and Biopharmaceutics, University of Bari, Bari, Italy

**Keywords:** pathogen latency, (re)activation, protein synthesis, cross-reactivity, codon usage, tRNAs, codon optimization

## Abstract

Infectious diseases pose two main compelling issues. First, the identification of the molecular factors that allow chronic infections, that is, the often completely asymptomatic coexistence of infectious agents with the human host. Second, the definition of the mechanisms that allow the switch from pathogen dormancy to pathologic (re)activation. Furthering previous studies, the present study (1) analyzed the frequency of occurrence of synonymous codons in coding DNA, that is, codon usage, as a genetic tool that rules protein expression; (2) described how human codon usage can inhibit protein expression of infectious agents during latency, so that pathogen genes the codon usage of which does not conform to the human codon usage cannot be translated; and (3) framed human codon usage among the front-line instruments of the innate immunity against infections. In parallel, it was shown that, while genetics can account for the molecular basis of pathogen latency, the changes of the quantitative relationship between codon frequencies and isoaccepting tRNAs during cell proliferation offer a biochemical mechanism that explains the pathogen switching to (re)activation. Immunologically, this study warns that using codon optimization methodologies can (re)activate, potentiate, and immortalize otherwise quiescent, asymptomatic pathogens, thus leading to uncontrollable pandemics.

## Introduction

Infectious pathogens—from viruses to fungi—establish latent infections in the human host and can, then, reactivate with severe pathologic sequelae. To quote some examples:

- Herpes simplex virus (HSV) types 1 and 2 that are capable of establishing lifelong infection primarily in neurons, and (re)activation of which may be accompanied by herpes encephalitis and recurrent vesicular eruptions in the orolabial and genital mucosa. 1 2 3
- Likewise, human herpesvirus 6A and 6B establish latency in the central nervous system, with potential to reactivate and cause multiple sclerosis and epilepsy, respectively. 4
- Human cytomegalovirus (HCMV) is a nearly ubiquitous β-herpesvirus capable of establishing a latent phase in humans. 5 6 HCMV (re)activation may associate with both systemic and end-organ severe diseases. 7 8 9
- Epstein–Barr's virus establishes and maintains latency in B cells, and its (re)activation may associate with several malignant tumors 10 11 and a vast number of pathologies. 12 13
- *Mycobacterium tuberculosis* is able to persist for the lifetime of the host, indicating that this pathogen has substantial molecular mechanisms to resist host-inflicted damage. Infection of humans with *M. tuberculosis* is frequent and can also lead to brain tuberculomas and meningitis. 14
- *Toxoplasma gondii* can remain dormant for years as bradyzoite within the host. 15 *Toxoplasma gondii* (re)activation may associate with chorioretinitis, encephalitis, and neuropsychiatric disorders such as schizophrenia. 16
- *Plasmodium falciparum* can reactivate during pregnancy after years of latency. 17
- *Cryptococcus neoformans* is a common central nervous system pathogen and causes fatal fungal meningoencephalitis, especially in immunocompromised subjects. 18 19

Pathologically, the disease burden related to pathogen (re)activation is overwhelming and eradication of chronic latent infections is a health top priority, especially when considering that latent infections are widespread in all over the world. 20 21 22 23 24 25 In general, persistent pathogen infections have been associated with an immune response that is unable to react with pathogen-infected cells. 26 In the years, escape from immune surveillance has been explained as possibly due to inhibition of host cell human leukocyte antigen class II expression 27 ; suppression of the expression of multiple genes that are important for antigen processing and presentation 28 ; selective elimination of Th-cells by apoptosis 29 ; escape from cytotoxic T lymphocytes 30 ; antigenic drift 31 ; production of immunosuppressive molecules 32 33 ; targeting of dendritic cell-specific intercellular-adhesion-molecule-3-grabbing nonintegrin 34 ; and hijacking of the lipoxygenase machinery of the host, 35 inter alia. On the whole, this corpus of data contributed important knowledge advancement of virology and microbiology, but unfortunately, the mechanism(s) underlying pathogen quiescence remain elusive. 36

Metabolically, it has been repeatedly observed that the pathogen persistence in the human host is characterized by restriction of pathogen protein production 37 so that passage from latency to (re)activation requires ex novo protein synthesis. 38 39 Then, it is assumed that chronic latent infections cannot be eradicated since, given the minimal expression of pathogen proteins, the host immune system cannot recognize the infected cell through the pathogen peptides presented on its cell surface. That is, antigenemia and patient immune responses are correlated, 40 41 by being the outcome of the antibody response: a question of antigen dose. 42 Simply put, in absentia of pathogen protein synthesis, there is no pathogen target that might evoke antipathogen attacks by the host immune system and, as a consequence, latent infections cannot be eradicated.

In contrast with this view and based on reports 7 8 12 13 documenting a high level of peptide sharing between pathogens and human proteins, the author's laboratory studies on cytomegalovirus (CMV) 43 44 analyzed the restriction of CMV protein synthesis as a device imposed via human codon usage purposely to block immune responses, with the ultimate aim of protecting the host from potential harmful autoimmune cross-reactions. 45 46 47 48 Indeed, lack of pathogen protein expression would prevent not only immune attacks against the pathogen proteins but would also inhibit cross-reactive autoimmune reactions against the host proteins sharing sequences with the pathogens. Expanding these studies, here the human codon usage has been compared with that of four genes coding for (re)activation-related proteins from HSV-1, *M. tuberculosis* , *P. falciparum* , and *C. neoformans* , respectively. Results document and confirm the role of the human codon usage in determining the silencing of pathogen protein expression, and highlight the correlation between codon frequencies and amounts of the corresponding isoaccepting tRNA as the biochemical mechanism that can trigger pathogen (re)activation.

## Methods

The gene coding sequences (open reading frames, ORFs) from the following four pathogen proteins were analyzed for codon usage:

- major viral transcription factor ICP4 (ICP4; UniProt: P08392, ICP4_HHV11, GenBank: AAA96675.1) from HSV-1 (NCBI:txid10298);
- transcriptional regulator WhiB5 (WhiB5; UniProt: P71592; WHB5A_MYCTU; GenBank: CCP42744.1) from *M. tuberculosis* (NCBI: txid83332);
- proliferation-associated protein 2 g4 (2 g4; UniProt: Q8ILI2_PLAF7; NCBI reference sequence: XM_001348399.1) from *P. falciparum* (NCBI:txid36329);
- eukaryotic translation initiation factor 3 subunit A (eIF3a; UniProt: P0CN42, EIF3A_CRYNJ; NCBI reference sequence: XM_570890.1) from *C. neoformans* (NCBI:txid214684).

The ORF of the human protein Sushi repeat-containing protein SRPX2 (SRPX2; UniProt: O60687; SRPX2_HUMAN; NCBI Reference Sequence: NM_014467.3) was analyzed as a control.

Codon usage of the *Homo sapiens* ORFeome was obtained from the international DNA sequence database ( http://www.kazusa.or.jp/codon/ ). 49 Codon usage of the ORFs coding for the above-listed proteins was obtained using GeneInfinity program ( http://www.geneinfinity.org ). Protein details were obtained from UniProt resource ( www.uniprot.org/ ). 50

## Results

### The Genetic Basis of Pathogen Latency

Four ORFs coding for proteins that are representative of viral, bacterial, protozoan, and fungal pathogens, respectively, were analyzed for codon usage. Results were compared with the codon usage of the human ORFeome. The four pathogen proteins were selected because of their crucial role in pathogen (re)activation, that is, specifically:

- HSV-1 ICP4 is a major viral transcription factor that is necessary for the transition from immediate early gene transcription to later viral gene transcription; 51
- WhiB5 is a transcriptional regulator that contributes to *M. tuberculosis* virulence and (re)activation; 52
- 2 g4 is a proliferation-associated protein that belongs to the proteases implicated in the *P. falciparum* erythrocytic replication cycle including merozoite egress from schizonts, host cell invasion by merozoites, and hemoglobin degradation; 53 54
- *C. neoformans* eIF3a is a subunit of the eukaryotic translation initiation factor 3 (eIF-3) complex. The eIF-3 complex specifically targets a subset of mRNAs involved in the cell proliferation. 55

In addition, the human protein SRPX2 was used as a control because it is expressed in the brain, an organ where pathogen quiescence preferentially occurs. In particular, SRPX2 is expressed in neurons of the rolandic area of the brain with a role in the perisylvian region, critical for language and cognitive development. 56

**Fig. 1:**
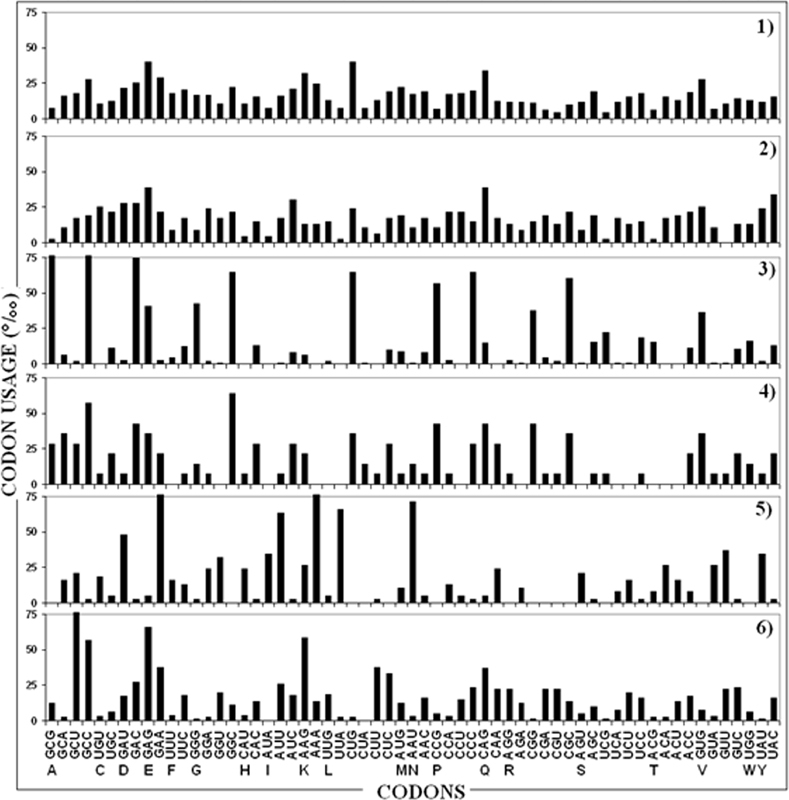
Codon usage of (1) human ORFeome, and ORF coding for: (2) human SRPX2; (3) HSV-1 ICP4; (4) *M. tuberculosis* WhiB5; (5) *P. falciparum* 2 g4; and (6) *C. neoformans* eIF3a. Codon usage is expressed as codon frequency per thousand. In the abscissa, amino acids given in one-letter code.

The comparative pathogen versus human codon usage pattern is illustrated in Fig. 1 and numerically tabulated in Supplementary Table S1 (online only).

Fig. 1 shows four fundamental points:

- All of the 61 codons that specify the 20 amino acids in the genetic code are used in the human ORFeome ( Fig. 1 , panel 1).
- The control, that is, the neuronal human SRPX2 ORF, conforms to the human ORFeome in the codon choices ( Fig. 1 , panel 2).
- In contrast, the four proteins derived from pathogens and essential for (re)activation 51 52 53 54 55 are coded by ORFs characterized by codon usage patterns markedly different from those of the human ORFeome and neuronal human SRPX2 ORF, with many codons being unused and a few codons being overused ( Fig. 1 , panels 3–6 vs. panels 1 and 2).
- The codon usages of the four ORFs coding for the pathogen proteins are strikingly different among themselves and have no codon choices in common and, rather, each of the four ORFs uses a highly specific codon pattern ( Fig. 1 , panels 3–6).

In sum, Fig. 1 shows that the usage of synonymous codons in ORFs that code for (re)activation-related pathogen proteins differs from the human codon usage. A striking example is the HSV-1 ICP4 ORF that preferentially uses the Ala codon GCG (76.98‰) that, instead, is rarely used in the human ORFeome as well as in the human neuronal SRPX2 ORF (7.37 and 2.15‰, respectively) ( Fig. 1 , panel 3 vs. panels 1 and 2, and Supplementary Table S1 ). Likewise, deviations from the human codon usage are evident in the bacterial, protozoan, and fungal ORFs coding for the (re)activation-related proteins analyzed here ( Fig. 1 , panels 4–6 vs. panels 1 and 2, and Supplementary Table S1 ).

Then, given the long-standing notion that codon usage is a basic determinant of gene expression, 57 58 59 60 61 62 results illustrated in Fig. 1 and tabulated in Supplementary Table S1 provide physical reality to the working hypothesis according to which deviation from the host usage of synonymous codons represents a powerful genetic constraint capable of blocking pathogen protein synthesis in the human host. In fact, as a documented known rule, 63 the ORFs/ORFeome of each biological entity—from proteins to proteomes, from viruses to humans—are characterized by specific sets of synonymous codons that determine/inhibit/modulate the protein expression pattern in cells, tissues, and organisms. Accordingly, ORFs that preferentially use optimal synonymous codons (i.e., the most abundant ones) are easily expressed, while ORFs that do not match with the host ORFeome and use nonoptimal synonymous codons (i.e., the rare ones) will be expressed at a very limited extent, if any.

### Biochemical Basis of Pathogen (Re)Activation

Data illustrated in Fig. 1 and Supplementary Table S1 locate the molecular basis of the lack of pathogen protein expression in the human host as due to different codon usages, but by themselves do not explain how pathogen usage of suboptimal codons can lead to a block of pathogen protein synthesis.

**Fig. 2:**
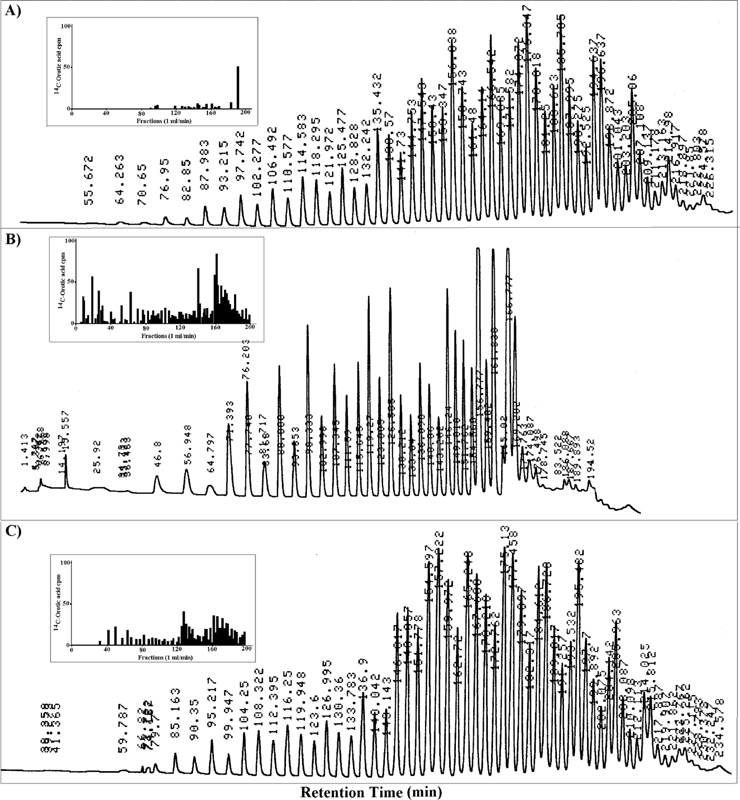
Changes of tRNA species during cell proliferation. High-performance liquid chromatography of (A) hepatic cytoplasmic tRNA population from quiescent nontreated, and from proliferating liver at (B) 20 hours and (C) 1 week following partial hepatectomy. In each panel, the inset reports [ ^14^ C]orotic acid incorporation into tRNA. (From Kanduc, 69 with experimental details in the reference.)

Actually, since 1980s, 64 65 66 67 68 it was demonstrated that, mechanistically, the basis for the correlation between rarely used codons and restricted protein expression (or, vice versa, highly used codons and high protein expression) resides in the quantitative matching between synonymous codons and isoaccepting tRNAs. That is, codon frequencies correlate with the amounts of the corresponding isoaccepting tRNA so that optimal, highly used codons correlate with abundant isoaccepting tRNAs, whereas rarely used, low-frequency codons correlate with low amounts of the corresponding isoaccepting tRNAs. Such a quantitative relationship between codons and isoaccepting tRNAs implies that the composition of the tRNA isoacceptor pools has to change in order pathogen protein expression can be resumed. In effect, changes in the composition of the tRNA isoacceptor pools occur under growth conditions. As documented in Fig. 2 , changes of tRNA isoaccepting species, as both relative percentage of total tRNA and absolute concentration, occur during cell proliferation induced by partial hepatectomy. 69

Specifically, Fig. 2 shows that tRNAs which are abundant under quiescence decrease during cell proliferation, and vice versa, so that cell proliferation provides a metabolic window for resumption of pathogen protein expression.

In this experimentally validated perspective, it assumes a crucial importance the fact that pathogen (re)activation is mostly associated with immunosuppressive treatments, 70 71 72 73 74 75 76 77 that is, with treatments that in general implicate administration of glucocorticoids. As a matter of fact, it is well known that glucocorticosteroids can induce cell proliferation 78 79 80 81 82 83 84 85 86 87 88 89 90 91 so that, consequently, it can induce proliferation-associated tRNA changes and favor pathogen protein expression and (re)activation. Therefore, in a clinical context, the present study might also help understand the pathogen (re)activation phenomenon in infected fetuses and newborns (i.e., in organisms growing rapidly) 7 and pregnancy, 17 as well as in subjects treated with glucocorticoids following, for example, transplant procedures. 75 77

## Conclusion

A leitmotiv of the research conducted in the author's laboratory since 2000 92 is that, following immune responses against infectious pathogens, the extremely high level of peptide sharing between human proteins and infectious agents 92 93 94 can cause harmful autoimmune cross-reactions and severe pathologies in the human host. 7 8 12 13 48 In this scientific context and using CMV as a research model, 43 44 data have been obtained in favor of the hypothesis that, to avoid cross-reactivity, expression of genes essential for viral (re)activation is purposely blocked because of a viral usage of synonymous codons different from that of the host. Here, the present study provides further evidences in favor of such working hypothesis by comparatively analyzing the human codon usage to that of four ORFs coding for (re)activation-related proteins derived, respectively, from HSV-1, *M. tuberculosis* , *P. falciparum* , and C. *neoformans* . Indeed, the data illustrated in Fig. 1 substantiate the concept that human codon usage is a main factor able to block pathogen protein expression in the human host, in this way avoiding potential immune response-associated cross-reactions and, consequently, allowing a pacific, unharmful, quiet coexistence between potentially dangerous pathogens and the human host.

In practice, pathogen-restricted protein synthesis emerges as a crucial protective phenomenon that avoids immune responses and the associated potential autoimmunity. Accordingly, resumption of pathogen protein synthesis by the fine tuning of the quantitative relationship between codons and isoaccepting tRNAs via cell proliferation ( Fig. 2 ), that is, under proliferative conditions determined by therapeutical treatments (such as glucocorticoids) or physiological cell proliferation (growth, pregnancy) may trigger pathogen virulence by evoking antipathogen immune responses able to cross-react with the host proteins.

So, as a logical conclusion, the genetic basis that specifically characterizes the human gene expression, that is, the human codon usage, has the value of a powerful first-line defense in the human innate immunity. Useless to say, clinically, the present study and conclusions invite to revise approaches currently used for managing infectious diseases and related pathologies. This is all the more so in light of the current (re)emerging infectious threats such as the severe acute respiratory syndrome-related coronavirus 2. 95 96
